# The Role of Autobiographical Story-Telling During Rehabilitation Among Hip-Fracture Geriatric Patients

**DOI:** 10.5964/ejop.v14i2.1559

**Published:** 2018-06-19

**Authors:** Paola Iannello, Federica Biassoni, Laura Bertola, Alessandro Antonietti, Valerio Antonello Caserta, Lorenzo Panella

**Affiliations:** a Department of Psychology, Università Cattolica del Sacro Cuore, Milan, Italy; b Department of Physical and Rehabilitation Medicine, Orthopedic Institute “Gaetano Pini”, Milan, Italy; Department of Psychology, Webster University Geneva, Geneva, Switzerland

**Keywords:** hip fracture, rehabilitation, self-narration, elderly

## Abstract

Hip fracture is one of the most common health care problems among elderly people. Literature shows that high self-efficacy expectations and positive affect are some of the key issues in functional recovery after hip fracture. The present investigation tested whether self-narration of such life-breaking event influences self-efficacy and depression during the process of rehabilitation. We designed a Self-Narration Journey (SNJ) to be administered during the in-hospital rehabilitation. In Study 1, we investigated the influence of SNJ on depression and perceived self-efficacy. Study 2 aimed to explore the effect of SNJ, depression, and self-efficacy on functional recovery of independence to perform daily activities during the rehabilitation process. The data showed that the Self-Narration Journey proved effective in increasing the perceived self-efficacy and in lowering the level of depression. The present work highlights a significant effect of the SNJ on the functional recovery process.

## Hip Fracture Among Elderly People

World population is ageing rapidly. Italy is the second country after Japan with the largest ageing population in the world, since 22% of the population is 65 years of age or even older (Iolascon, Cervone, & Gimigliano, 2011). As people live longer, the number of age-related health problems is rising exponentially. Among others, hip fracture rates constantly increase in the ageing population (Chang, Center, Nguyen, & Eismen, 2004; Ishizaki et al., 2004; Johnell & Kanis, 2004). Hip fracture is one of the most common and serious health care problems among elderly people. Indeed, hip fractures are a major cause of mortality and disability, given that the risk of dying within the first year after this injury has been reported to be as high as 13-36% (Johansen, Mansor, Beck, Mahoney, & Thomas, 2010; Pretto et al., 2010).

Hip fractures involve remarkable medical and rehabilitation costs (Marks, Allegrante, MacKenzie, & Lane, 2003), requiring an average hospital stay of 19-24 days. It is fair to say that hip fractures not only involve a broken bone, but also have a significantly psychological effect. A number of studies, in this respect, have found that hip fracture is frequently associated with negative emotional status, low self-confidence, and depression (Barangan, 1990; Holmes & House, 2000; Zimmerman et al., 1999). Some of these studies highlighted that hip fracture patients tend to express feeling of insufficiency and insecurity, and a general loss of self-confidence, specifically related to the difficulties they encounter in executing even trivial daily activities (Zidén, Wenestam, & Hannsson-Scherman, 2008). Other studies, additionally, tried to establish a connection between depression symptoms and rehabilitation outcome, suggesting that depression might interfere with the patients’ engagement in rehabilitation process (Feinstein, 1999; Lenze et al., 2004).

Given both the prevalence (Reginster, Gillet, & Gosset, 2001) and the serious consequences – both physical and psychological- of hip fractures in elderly people, there is a strong need to examine all factors that might improve current hip fracture rehabilitation strategies and recovery.

Many factors are reported to affect the outcome of the patients’ post-operative rehabilitation in terms of both functional recovery and the quality of life. Some factors specifically concern the medical domain, such as early surgical time (Schürch et al., 1996), pre-fracture functional abilities, and comorbid conditions (Fried, Ferrucci, & Darer, 2004). However, social and psychological rather than physical factors are likely to affect rehabilitation (Repper & Perkins, 2003; Roberts & Wolfson, 2006). Indeed, literature indicates that patients’ psychological characteristics could play an important role during the rehabilitation process (Givens, Sanft, & Marcantonio, 2008). Specifically, being resilient (Rebagliati et al., 2016; Sciumè et al., in press) as well as developing high self-efficacy expectations (Allegrante, MacKenzie, Robbins, & Cornell, 1991) and positive affect (Fredman, Hawkes, Black, Bertrand, & Magaziner, 2006) turned out to be some of the key issues in functional recovery after hip fracture. Few studies have examined the ways in which elderly cope with hip fracture and the meaning that they give to such an event (Archibald, 2003; Hunt & Stein, 2004; Zidén et al., 2008). Recovery expectations are strictly related to the meaning the patients assigned to the experience of hip fracture. These findings suggest that to maximize the rehabilitation potential, a multidisciplinary effort that would consider not only physical, but also psychological factors is required.

## Self-Narration for Well-Being

In recent years, health care services have embraced the psychological dimension thanks to different models, including the narrative medicine approach. Narrative medicine offers a model for improving communication between patients and physicians, which in turn affects the patient’s well-being as well as the quality of care and health outcomes (Charon, 2008; Riva et al., 2014; Riva, Iannello, Antonietti, & Pravettoni, 2015). It allows a clinician to listen to the patient’ story; his or her fears, suffering, and hopes; and his/her description of how illness and medical care have affected his/her life. Regarding the patient-physician relationship, narrative medicine facilitates the creation of therapeutic alliances and the selection of treatments that make sense within the story told by patients and their families (Cepeda et al., 2008; Smorti, Pananti, & Rizzo, 2010).

The link between self-narration and well-being has been maintained in different fields. Psychoanalytic tradition has claimed that the “self-narration” processes that occur in psychoanalytic sessions have a therapeutic value because they stimulate insight, reformulation of one’s life history, and attribution of a new structure to life events (Schafer, 1992; Spence, 1982; White, 1990). The cognitive and the narrative approaches have emphasized that narration operates as an active “rebuilder of experiences”, allowing the individual to set events in a semantic plot line and endow them with narrative organization (Antonietti, Confalonieri, & Marchetti, 2014; Biassoni, Ciceri, & Antoniotti, 2005; Colombo, Iannello, & Antonietti, 2010; Conway, 2005). As Syrjälä, Takala, and Sintonen (2009) pointed out, narratives, according to Ricoeur (1991), mediate three specific relationships, those between a human being and his/her perception of the world (worldview), between a human being and another human being (communication), and between a human being and him/herself (self-understanding). Following Bruner (1987), storytelling is one of the primary mechanisms through which humans make sense of their experiences. In particular, telling the story of stressful or traumatic experiences can help develop a sense of understanding and control (Koenig Kellas, Trees, Schrodt, LeClair-Underberg, & Willer, 2010). Indeed, constructing stories facilitates a sense of resolution, which results in less rumination, whereas painful events that are not structured into a narrative format may contribute to the continued experience of negative thoughts and feelings. In his works, Pennebaker (1993, 1997, 2004) showed that patients deal with traumatic events more effectively if they can understand and assimilate them. When this happens, individuals no longer need to actively inhibit or hold back their thoughts and feelings. Indeed, several studies have indicated that actively inhibiting ongoing behavior is associated with both short-term autonomic activity (Fowles, 1980) and long-term stress-related disease. Confronting a trauma may thus reduce the long-term work of inhibition, since individuals may assimilate, reframe, or find meaning in the event (Horowitz, 1976; Silver, Boon, & Stones, 1983).

Because of its sense-making function, storytelling is potentially beneficial for individuals’ health and well-being (Neimeyer & Levitt, 2000). In fact, research in narrative psychology (e.g., McAdams, 1993; Pennebaker, 1997) and narrative therapy (e.g., Monk, 1997; White, 2007) suggests that the opportunity to tell and/or reframe stories of trauma, difficulty, or stress can have positive effect on mental and physical health (Koenig Kellas et al., 2010). Consistently, narrative medicine aims to empower patients by giving them a sense of control (Cepeda et al., 2008).

The ways in which individuals tell their stories affect the reframing process. Two factors that empower the effectiveness of the narration process have been identified. The first factor involves sense making as an interactional collaborative process while the other factor concerns the emotional content of narratives. Regarding the first factor, though research is needed to increase the knowledge of the ways in which dialogic narration affects the personal well-being, some promising data show that narrating personal experience to others prompts joint processes of sense-making co-construction of stressful events (Sales & Fivush, 2005; Trees & Koenig Kellas, 2009). Therefore, sense making emerges as the result of a dialogic construction. Concerning the second factor, as mentioned previously, telling the story of a traumatic or stressful experience allows an individual to express emotions and/or cognitively make sense of the trauma, which in turn permits the individual to “let go” of the memory and move on from potentially unhealthy ruminations. Research by Ramírez-Esparza and Pennebaker (2006) showed that individual narratives that are richer in emotional and insight language were related to better health outcomes. Consistently, a study by Thomas et al. (2009) on the narrative research methods in palliative care pinpointed that the more effective interviews focused on the following themes: cancer diagnosis and the course of the illness; experiences of medical treatments, care systems, and health care practitioners; the effect of the illness on the lives of self and others; the meanings, emotions, and practical challenges tied up with having cancer; and sources of support.

## The Present Investigation

The existing literature shows that exploring the patients’ perspective of the experience of hip fracture might positively affect the rehabilitation process (Archibald, 2003; Fredman et al., 2006; Zidén et al., 2008). The present investigation aimed to verify the existence of a connection between self-narration of such life-breaking event and some psychological dimensions that are relevant to the rehabilitation process.

To do so, we designed the Self-Narration Journey (SNJ) to be administered by a psychologist during the in-hospital rehabilitation and assessed its effects on some psychological variables. In particular, in Study 1, we investigated the influence of SNJ on depression and perceived self-efficacy in elderly patients who were undergoing the rehabilitation process. Study 2 aimed to explore the effect of SNJ, depression, and self-efficacy on functional recovery of independence in performing daily activities during the rehabilitation process.

## Study 1

### Method

#### Participants

Participants were orthopedic rehabilitation patients aged 60 and older who were hospitalized between September 2015 and December 2015 in the rehabilitation department of the Orthopedic Institute “Gaetano Pini” in Milan. All patients who underwent hip fracture surgery and followed their in-hospital rehabilitation program were considered candidates for the inclusion in the study. An initial screening assessed the level of cognitive functioning. Eight patients who scored <30 on the Mini-Mental State Examination (MMSE) (Bellelli, Frisoni, Turco, & Trabucchi, 2008) were excluded from the study. Of the initial 60 patients, 52 received written and verbal information about the study and were asked if they were willing to participate. Ten patients declined. Thus, finally size sample was of 42 (30 women and 12 men, age range: 60-95 yrs.; mean age = 79.67 yrs.; *SD* = 9.03). Patients were randomized to the intervention (*n* = 21; mean age = 79.95 yrs.; *SD* = 8.93) or usual care (control) group (*n* = 21; mean age = 79.31 yrs.; *SD* = 9.12). No socio-demographic differences were revealed between the patients who were randomized to the intervention and control group.

The Ethics Committee of the Orthopedic Institute “Gaetano Pini” approved the study. Informed consent was obtained from all patients during their rehabilitation stay.

#### Procedure and Materials

All patients underwent physical therapy rehabilitation sessions twice a day, six days a week throughout their in-hospital rehabilitation stay. The rehabilitation training included strengthening exercises and transfer, postural, and gait training. Upon admission (within 5 days after admission) and at discharge (within 5 days prior to discharge), a trained psychologist administered the following questionnaires to both intervention and control groups.

*Geriatric Depression Scale* (GDS; Sheikh & Yesavage, 1986; Italian version: Pedrabissi & Santinello, 1991). The GDS is a 15-item self-reported depression assessment scale (e.g. “Do you feel that your life is empty?”), which can be used with both healthy and medically ill older adults. Scores range from 0 to 15, with scores > 5 that are considered as suggestive of depression and scores ≥ 10 as almost always indicative of depression. It has to be noted that the validity and reliability of the short form of GDS have been supported through a validation study comparing the long and short Forms of the GDS for self-rating of symptoms of depression, both were successful in differentiating depressed from non-depressed people (*r* = .84, *p* < .001) (Sheikh & Yesavage, 1986).

*General Self-Efficacy Scale* (GSES; Sibilia, Schwarzer, & Jerusalem, 1995). The GSES includes 10 items measuring global feelings of self-efficacy (e.g. “I am confident that I could deal efficiently with unexpected events”). The responses are rated on a 4-point scale ranging from 1 (not at all true) to 4 (completely true). The total score is calculated by finding the sum of all the items. For the GSE, the total score ranges between 10 and 40, with a higher score indicating higher self-efficacy.

Only the intervention group completed the *Self-Narrative Journey (SNJ*; the entire SNJ interview is reported in the Appendix). The SNJ consists of a semi-structured interview designed specifically for the present study, with the aim to help the patient integrate the critical event of hip fracture in her/his autobiographical story and to support her/him in the process of sense-making about such a life-breaking event. The proposed format includes open-ended questions, asked in a fixed set. It has been chosen to attain two main aims: on the one hand, to foster free expression of feelings and emotions within a frame able to contain them; on the other hand, to drive the subject along a journey for meaning construction, since what has been expressed can find a placement in the narrative structure, featured by temporal and causal links (Biassoni et al., 2005; Bruner, 1987). The SNJ is divided into three main content sections, which correspond to the past, present, and future parts of a patient’s life. The *past* section focuses on the patient’s life before the hip fracture, such as her/his family, friends, and daily routine. The *present* section addresses the hip fracture event and the rehabilitation process, in particular the hospital daily routine and the relationship between the patient and both physicians and other patients. In the future section, the focus is on the patient’s perceptions and expectations of returning home. The patient is encouraged to reflect on possible solutions once he/she would realize that he/she would not be as autonomous and independent as before the hip surgery. The SNJ ends with two feedback questions. First, the patient is asked to rate how much he/she liked the interviews on a Likert scale ranging from 1 (not at all) to 5 (very much). The entire SNJ interview is reported in the Appendix.

The SNJ was adapted specifically for the elderly population. It was divided into five sessions, each comprising five to ten questions assessing the past, the present and ending with the future. Each part, administered during a meeting, took 15 to 20 minutes to complete in order to avoid the risk of an excessive load (both cognitively and emotionally) for the patient. Consequently, the number of meetings varied from three to five, depending on the patient’s willingness to narrate his/her story. Overall, the SNJ lasted for two weeks.

**Figure 1:**
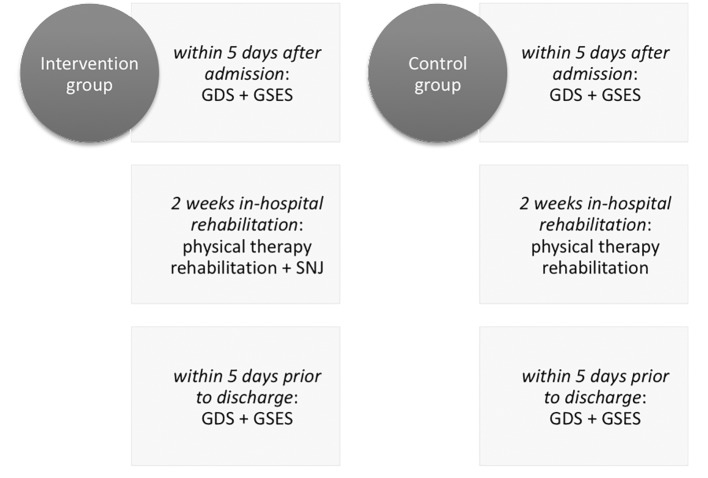
Design of Study 1.

The design of the study is illustrated in Figure 1.

### Results

Statistical analyses of all data were performed with SPSS version 23.0.

#### Preliminary Analyses

Before comparing depression and self-efficacy scores with respect to group (intervention vs. control) and time (at admission vs. discharge), the normality of distributions of the variables was assessed by calculating skewness and kurtosis values. Both values were within the +1 to -1 range, indicating that scores on both scales were normally distributed (Marcoulides & Hershberger, 1997).

The internal consistency of the scales was examined by calculating Cronbach’s alpha reliability coefficients, which showed good internal consistency (GDS: α = .89; GSES: α = .83).

#### Descriptive Analyses

Descriptive statistics for depression and self-efficacy scores under all conditions (intervention vs. control group and admission vs. discharge) are reported in Table 1.

**Table 1:** Means and Standard Deviations of GDS and GSES Under All Conditions

Scale	Intervention group				Control group			
	Admission		Discharge		Admission		Discharge	
	*M*	*SD*	*M*	*SD*	*M*	*SD*	*M*	*SD*
GDS	5.18	2.44	3.59	2.42	5.05	2.09	6.38	3.47
GSES	3.41	0.75	3.86	0.56	3.27	0.74	3.16	0.61

Values are comparable to the scores recorded in previous studies involving samples of patients similar to those engaged in the present study (Rebagliati et al., 2016; Sciumè et al., in press).

#### Influence of SNJ on Perceived Self-efficacy and Depression

A mixed multivariate analysis of variance (MANOVA) with time (at admission vs. discharge) as the repeated factor and group (intervention vs. control) as the between-subject factor was conducted on GDS and GSES scores.

A significant main effect of group (*F*(1,41) = 4.66, *p* < .05; Wilk's Λ = .89, partial η^2^ = .28, observed power = .82) and a significant interaction effect of group x time (*F*(1,41) = 12.901, *p* < .01; Wilk's Λ = .93, partial η^2^ = .25, observed power = .92) emerged.

**Figure 2:**
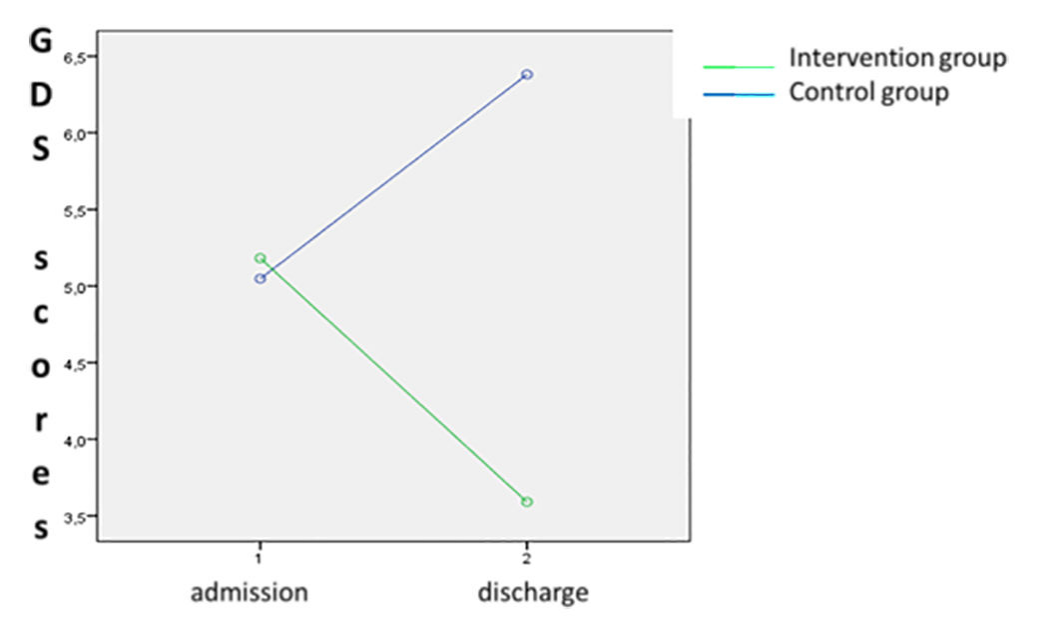
Interaction effect of group x time on GDS scores.

**Figure 3:**
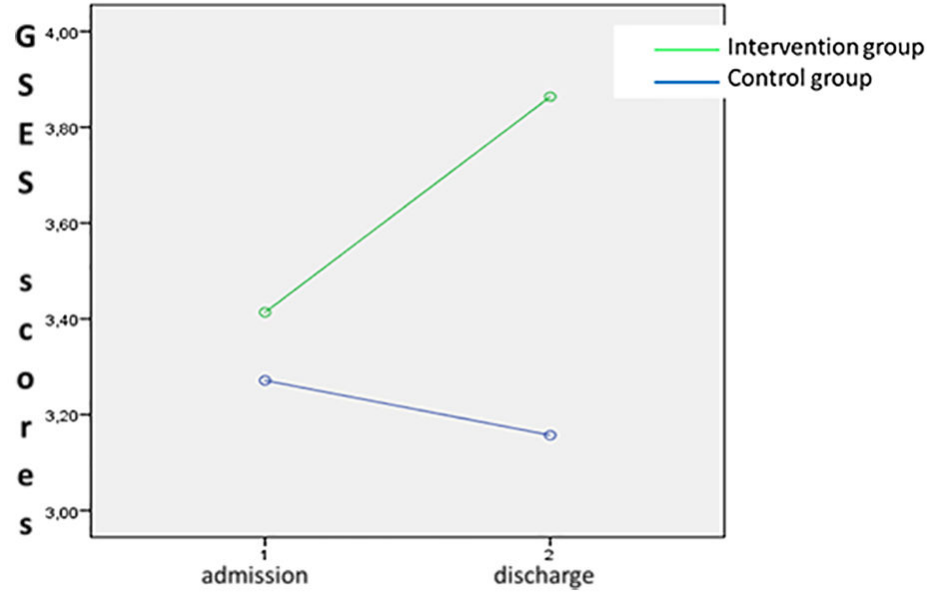
Interaction effect of group x time on GSES scores.

Specifically, the interaction effect of group x time was significant for both GDS scores (*F*(1,41 = 5.72, *p* < .05; partial η^2^ = .01, observed power = .84) and GSES scores (*F*(1,41= 3.74, *p* < 05; partial η^2^ = .14, observed power = .82) (Figures 2 and 3).

## Study 2

### Method

#### Participants

Participants were 40 orthopedic rehabilitation patients (30 women and 10 men: age range = 68-92 yrs.; mean age = 80.12 yrs; *SD* = 7.09) who were hospitalized between February 2016 and June 2016 in the rehabilitation department of the Orthopedic Institute “Gaetano Pini” in Milan after undergoing hip fracture surgery.

As in Study 1, cognitive status was initially assessed to ensure that only patients with a score >30 at the MMSE are included in the research (12 patients who scored <30 were excluded).

After the initial screening, patients were randomized to the intervention (15 women and 5 men; mean age = 80.00 yrs.; *SD* = 7.19) or control group (15 women and 5 men; mean age = 80.25 yrs., *SD* = 7.20) conditions.

The Ethics Committee of the Orthopedic Institute “Gaetano Pini” approved the study. Informed consent was obtained from all patients during their rehabilitation stay.

#### Procedure and Materials

The procedure was the same as described in Study 1.

In Study 2, upon admission and at discharge, both intervention and control groups were administered the Geriatric Depression Scale (GDS) and the General Self-Efficacy Scale (GSES), as in Study 1. In addition, the two groups were administered the questionnaires described as follows.

*Barthel Activities of Daily Living Index* (BI; Mahoney & Barthel, 1965) is one of the most widely used rating measures of activity limitations in patients with neuromuscular and musculoskeletal conditions in rehabilitation settings. BI is a 10-item scale that measures functional independence in the domains of personal care and mobility. Specifically, the scale assesses the following personal activities, feeding, personal toileting, bathing, dressing, toilet transfer, controlling bladder, controlling bowel, wheelchair transfer to and from bed, walking, and ascending and descending stairs. Each item is rated in terms of whether the patient can perform the task independently, with some assistance, or is dependent on help based on observation (0 = unable to perform the task, 5 = help needed 10 = independent). An overall score is computed by summing the rating values. Scores range from 0 to 100, in steps of 5, with higher scores indicating greater independence.

*Psychological Well-Being Scale (PWB;* Ryff & Keyes, 1995; Italian validated version: Ruini, Ottolini, Rafanelli, Ryff, & Fava, 2003). The PWB, which was administered to both groups of participants only on admission, consists of 84 questions reflecting 6 distinct components of psychological well-being, the individual sense of self-determination (Autonomy), the capacity to manage effectively one’s life and surrounding world (Environmental Mastery), a sense of growth and development as a person (Personal Growth), the possession of quality relations with others (Positive Relations with Others), the conviction that one’s life is purposeful (Purpose in Life), and a positive evaluation of oneself (Self-Acceptance). The respondents rated statements on a scale ranging 1 to 7, with 1 indicating strong disagreement and 7 indicating strong agreement.

**Figure 4:**
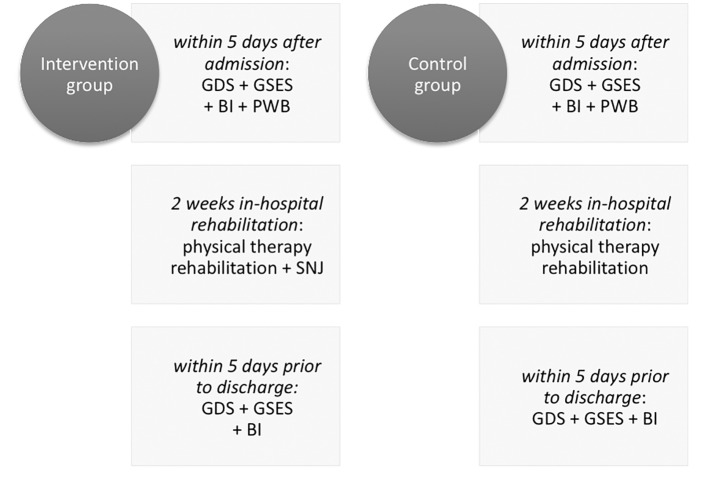
Design of Study 2.

Figure 4 illustrates the design of this study.

### Results

#### Preliminary Analyses

Normal distribution of the scores on all scales was assessed by calculating skewness and kurtosis values. Both skewness and kurtosis were within the +1 to -1 range, indicating that scores were normally distributed (Marcoulides & Hershberger, 1997).

Cronbach’s alpha reliability coefficients were computed for all scales. The coefficients for the first two scales (GDS: α = .90; GSE: α = .83) were similar to those computed in Study 1, supporting the internal consistency of the instruments. BI (α = .88) and all of the PWB subscales showed good levels of internal consistency (Autonomy: ɑ = .77; Environmental Mastery: ɑ = .73; Personal Growth: ɑ = .73; Positive Relations with Others: ɑ = .79; Purpose in Life: ɑ = .77; Self-Acceptance: ɑ = .83).

Prior to the regression analysis, the patients’ level of psychological well-being in both intervention and control groups was assessed to verify that no significant differences existed between the two groups upon admission. T-tests for independent samples were performed on all PWB dimensions to compare intervention and control groups. The results indicate that the two groups did not differ in PWB subscales (Autonomy: *t* = 1.65, *p* = .11; Environmental Mastery: *t* = 0.83, *p* = .41; Personal Growth: *t* = 0.76, *p* = .45; Positive Relations with Others: *t* = 0.99, *p* = .33; Purpose in Life: *t* = 1.42, *p* = .17; Self-Acceptance: *t* = 0.51, *p* = .62).

#### Influence of SNJ, Depression and Self-Efficacy on Functional Status at Discharge

A regression model was performed to assess the influence of demographic, physical, and psychological factors on the functional status at discharge, which was operationalized as the difference between BI at discharge and BI on admission (∆ BI).

Demographic and functionality predictors (age and BI on admission) were entered in a hierarchical linear regression first, followed by all other psychological predictors (SNJ in the first step and depression and perceived self-efficacy in the last step). Table 2 displays the standardized regression coefficients (ß) and R-square after entering all independent variables.

**Table 2:** Hierarchical Linear Regression With ∆ BI at Discharge as the Dependent Variable and Age, BI on Admission (First Block), Group (Intervention vs. Control) (Second Block), Depression and Self-Efficacy (Third Block) as Independent Variables: The Standardized Regression Coefficients (ß) and R-Square

Predictor	ß
1. Age	-.33 (*p* = .056)
2. BI on admission	-.51 (*p* < .005)
3. Group	-.25 (*p* = .060)
4. Depression	-.38 (*p* < .050)
5. Self-efficacy	.30 (*p* < .050)

*Note.* First block (demographic/physical variables): *R*^2^ = 0.122; *F*(2,39) = 5.27, *p* < .05. After entering the second block (group): *R*^2^ = 0.250; *F*(3,39) = 3.59, *p* < .05. After entering the third block (depression/self-efficacy): *R*^2^ = 0.47; *F*(7,39) = 4.04, *p* < .005.

The regression model, including all independent variables, was statistically significant (*R*^2^ = 0.47; *F*(5,39) = 4.04, *p* < .005), indicating that a linear combination of the predictors included in the regression model accounted for 47% of the variation in functional status.

After entering the psychological predictors in the regression analysis, the variance accounted for in the level of functional status significantly improved, beyond that afforded by the demographic and physical variables. In particular, the BI on admission, and both the level of depression and the perceived self-efficacy turned out to be significant predictors of functional status. Moreover, the results indicated that the training (SNJ) had an influence on the functional status at discharge although it did not reach the significance level.

## General Discussion and Conclusions

The SNJ is based on autobiographical narration and administered it to a sample of older patients during in-hospital rehabilitation after hip fracture surgery to test its influence on the patients’ well-being and functional recovery. Since hip fracture not only involves a broken bone, but also has an enormous psychological effect that has to be considered in health care, the training has been developed to improve the overall recovery outcomes. We aimed to verify whether exploring the perspective of patients on such a life-breaking event and providing them – through self-narration – with a space for sense-making activity might improve their perceived self-efficacy, decrease their depression level, and thus empower their personal well-being. Moreover, we aimed to verify whether increasing the patients’ well-being affects their functional recovery.

Literature on hip fracture indeed provides evidence that such experience significantly affects the relation patients have with their own body, themselves, other people, and their life situation. The feelings of losing confidence in their body; being limited in movements and therefore depending on others; staying secluded at home; feeling disabled, older, and closer to death; as well experiencing pain and loss of control, autonomy, and self-efficacy are possible triggers of depression (Archibald, 2003; Zidén et al., 2008). The research shows that patients with high positive affect respond more effectively and faster to functional recovery compared to patients with low positive affect and depressive symptoms (Fredman et al., 2006). In addition, a work by Allegrante and colleagues (1991) underlined the role of self-efficacy in modifying the outcomes of recovery in the specific case of rehabilitation interventions after hip fracture and surgery.

For these reasons, in Study 1, we focused on the possible effects of the narrative training on self-efficacy and depression, usually investigated in relation to hip fracture and the subsequent rehabilitation process. Supported by Study 1’s results, in Study 2, we broadened the range of the considered variables, including the Barthel ADL (Activities of Daily Living) index, as a measure of functional recovery, recorded before and after the rehabilitation process.

The results of the two studies were concordant. Data collected in Study 1 showed that the self-narration journey proved effective in increasing the perceived self-efficacy and in lowering the level of depression. The data collected in Study 2 replicated these finding, showing that the autobiographical story telling enhanced the perception of self-efficacy and had a positive effect on the level of depression. Moreover, Study 2 revealed a significant effect of the proposed training on the functional recovery process. Patients in the experimental group who took part in the autobiographical training reported higher Barthel ADL index scores at the end of the rehabilitation process. Therefore, the data collected in the present work are consistent with literature, which has shown that the sense-making function makes storytelling potentially beneficial for individual health and well-being (Neimeyer & Levitt, 2000).

As said, narration operates as an active “rebuilder of experiences,” allowing the person to set events in a semantic plot line and defining meaningful causal connections between various events and between events and emotional experience (Conway, 2005; Schafer, 1992). Re-narrating a traumatic event helps the individual integrate it in his or her individual history to develop a sense of understanding and control (Koenig Kellas et al., 2010). Finally, narrative activity enhances self-reflection. Tracing meaningful connections between events and emotional reactions helps the individual increase his/her self-efficacy (Chamberlain & Haaga, 2001).

Possibly, the described sense-making activity also provides individuals with the sense of increasing control of an event, like hip fracture, experienced as an abrupt and uncontrollable event with considerable consequences. Unlike many illnesses and diseases, which bring about the need to confront death, hip fracture represents physical disease, disability, accident, and trauma, and requires copying with a new image of the self that is more fragile, less efficient, and more dependent on others. Facing such new image can give origin to feelings of loss, depression, and anxiety. The possibility to share those feelings with someone who is willing and competent to accept them is likely to increase positive affect while reducing the negative one. Telling the story of a traumatic experience allows an individual to express his or her own emotions and cognitively make sense of the trauma, which in turn allows the individual to “let go” of the memory and move on from potentially unhealthy ruminations.

Besides, receiving acceptance, legitimateness, and confirmation also increases the sense of acceptance of both the event and the subject’s own reactions. Indeed, constructing stories facilitates a sense of resolution, which decreases rumination as well as negative thoughts and feelings that being replaced by the feeling of continuity of own life. Hence, the life-breaking event of hip fracture can find a meaning along a comprehensible track. Therefore, the event itself can be interpreted in the light of its connections with the previous and consequent condition, turning from an event that represents “the end” into a change that challenges the individual to find new life strategies in the frame of a possible personal growth.

The work presented here has some limitations. The small sample size could limit the generalizability of the findings. Moreover, collecting the data 6 months after the discharge would add further evidence of the effectiveness of SNJ in improving the perceived self-efficacy and in regulating the depression level across a longer period.

The studies also have some strengths. First, we adopted an integrated perspective, underlining the link between psychological wellbeing and physical and functional recovery processes. From such a perspective, the described intervention has been designed to improve the overall recovery process with an aim to lower both the individual discomfort and the public cost of health care. The integrated perspective on well-being also chased from the methodological viewpoint, as we collected the data on both physical and psychological well-being and functionality. Second, although plenty of literature has investigated the power of autobiographical narration in improving the individual well-being, to our knowledge, quantitative data about such effect collected through experimental methodologies are lacking. The present work represents an attempt to bridge a gap in this direction.

Finally, the SNJ is an economic and undemanding tool, since it requires involvement of a single professional who can be easily and briefly trained. Moreover, the SNJ is a non-intrusive practice that could be easily incorporated into the ordinary ward routine.

## Appendix: Self Narrative Training

### Session 1

Introduction

“Good morning. Today I would like to retrace some moments of your past with you. We are going to talk about your current situation and your perceptions of your future.”

Thematic Area: The Past

- PAST 1 – SOCIO-DEMOGRAPHIC PROFILE
  Let's start from the beginning of your personal story ...
  - When and where were you born?
  - Where do you live now?
  - What was your job?
- PAST 2 – FEELINGS
  Now I would like to retrace your emotional past …
  - Who are the most important people in your life?
  - How would you describe your relationship with them?
  - What did/do you enjoy about the relationship with them?
  - What did/do you not enjoy about the relationship with them?
- PAST 3 – HAPPY LIFE EVENT
  - Would you describe a happy episode of your life?

### Session 2

- PAST 4 – BEFORE THE HIP FRACTURE
  Think about your life before the hip fracture ...
  - Would you describe your typical daily routine before the hip fracture?
  - What did you like to do in your spare time? What were your hobbies and interests?

Thematic Area: The Present

- PRESENT 1 – HIP FRACTURE
  Today, we are going to speak about the hip fracture event …
  - Where and how did it happen?
  - How did you feel immediately?
  - What was your reaction? Were you alone? Who did you call for help?

### Session 3

- PRESENT 2 – ARRIVAL AT THE HOSPITAL – AFTER THE INTERVENTION
  - How did you arrive at this hospital? Who was together with you? How have you been welcome in this hospital?
  - How did you feel at your arrival? Did you feel accepted or neglected?
  - How did you feel during your first day in this hospital?
- PRESENT 3 – TYPICAL DAILY ROUTINE IN THE HOSPITAL
  Now I would like you to tell me about your daily routine in the hospital ...
  - Please define your mood while staying here in the hospital.
  - Are you experiencing any difficulties here in the hospital?
  - Do you miss someone or something?
  - Is there any positive or pleasant aspect?
  - Do you have any recurring thoughts?

### Session 4

- PRESENT 4 – RELATIONSHIPS WITH PEOPLE IN HOSPITAL
  We will now focus on the relationships you are experiencing here in hospital …
  - Tell me about your relationship with your roommate.
  - Have you known/become friends with patients staying in other rooms?
  - Tell me about your relationship with doctors and nurses.
  FOCUS ON THE DOCTOR-PATIENT RELATIONSHIP
  - Do you like your doctor? What do you like and what don’t you like?
  - What do you think of the prescriptions and advice you received from your doctor?
  - On a scale from 1 to 10, how much do you trust your doctor?
  - Which aspects are important, in your opinion, to trust a doctor? Why?
- PRESENT 5 – ADVICES
  - Give an advice to a person who is in the same situation as you. What would you say?
  - What advice would you give to yourself?
  - What are your resources, your strengths, and your weaknesses?

### Session 5

Thematic Area: The Future

- FUTURE 1 – BACK HOME
  Let us think about your future …
  - How do you imagine your return to home?
  - Do you think your life will change after the period you stayed in hospital?
- FUTURE 2 – CLOSING
  At your discharge ...
  - Do you think you will need assistance or are you going to be autonomous?
  If you feel you will need assistance ...
  - Who would you ask for help/support?
  - If you had a magic wand, what service would you need at home to feel more secure?

Feedback Questions

Please rate the pleasantness of this experience of training.

**Table:**

1	2	3	4	5
Not at all				Very much

Please rate the usefulness of this experience of training.

**Table:**

1	2	3	4	5
Not at all				Very much

Thank you.
